# Safety Influencing Factors and Management Countermeasures of Patients Transferred from ICU in Transition Period Based on Intelligent Processor Three-Dimensional Quality Model

**DOI:** 10.1155/2022/1455830

**Published:** 2022-01-25

**Authors:** Ping Huang, Li Zhu, Qi Wu, Weishu Hu

**Affiliations:** ^1^Critical Care Medicine, Chongqing People's Hospital, Chongqing 400013, China; ^2^Department of Hepatobiliary and Pancreatic Surgery, Chongqing People's Hospital, Chongqing 400013, China

## Abstract

With the development of science and technology of the times, the level of medical care is constantly improving. For patients transferred from ICU, the intelligent processor 3D quality model technology has gradually played an important role in clinical treatment and has become a new type of attention. In order to understand the implementation status of transitional care and the feelings of transitional care for patients transferred from ICU and understand the views of transitional care-related department doctors on transitional patient care and the role that the intelligent processor three-dimensional quality model can play, this article passed a review of the city ICU transferred patients from a hospital that conducted related investigations, reviewed related literature, conducted interviews with professionals, etc., collected relevant information, constructed case templates, and created a clinical research model using comprehensive quantitative and qualitative analysis methods. The results of the study found that, after treatment, patients transferred from the ICU based on the intelligent processor's three-dimensional quality model have higher physical activity than patients treated by other methods, the ratio is more than 20%, and the postoperative recovery efficiency of patients is higher than 15% and more. This shows that the three-dimensional quality model based on the intelligent processor can improve the important role in the transition period of patients transferred from ICU.

## 1. Introduction

In the transitional care model (TCM) due to changing diagnostic and therapeutic environment and care needs, patients need to be transferred between different levels of care if the patient is in the treatment and recovery stages [1]. Transitional care is usually allocated during the transition period to ensure patient coordination and continuity of nursing work during the transition period and to accept various corresponding nursing behaviors. Transition theory defines “transition” as the transition or movement from one state, situation, or position to another state or position [2].

The quality of care services for patients transferred from the ICU will be affected by the transitional care provider: the hospital nurse. Since ICU patients usually have complex medical problems, the transitional environment for ICU patients is not only the intensive care unit, the community, and the family, but covers also other sectors, such as health, rehabilitation, and ENT services [3]. The opinions and feelings of participants and witnesses are one of the criteria for evaluating the quality of care during the transition period. During the transition period, nurses, patients, and their families have different social roles, influencing each other and benefiting each other. In the medical environment, the internal experience of the patient's family is influenced by the patient's illness and the external environment. At the same time, the nurse is the main medical staff contacting the patient's family [4]. After being discharged from the hospital, the nurse is considered a mediator in establishing family autonomy in patient care. Their subjective emotions and experiences will affect the quality of nursing services and indirectly affect the psychological emotions of the patient's family. The subjective emotions of these different groups of people permeate and relate to each other. Recognizing the subjective feelings of patients' family members, clinicians in relevant departments, community nurses, and doctors, and providing inspiration for the integrated implementation of transitional care, can directly or indirectly improve the satisfaction of patients who transfer from ICU to nursing service department.

For postoperative care of patients, domestic and foreign experts also have many studies. Cooper and others believe that family participation in nursing is an important part of ICU. FCC demonstration overseas implementers are parents, nurses, respiratory therapists, facilitators, and social work physiotherapists, children's life experts, and a multiprofessional team including pharmacists, focusing on home care. Many foreign hospitals have begun to experiment with new management systems, including public visits, where family members can freely choose whether to accept other hospital support and other measures [5]. Tully was to determine the point prevalence rate of medication errors during the transition from ICU to non-ICU location and to evaluate the types of errors and risk factors for medication errors during the transition period of care. A 7-day point prevalence study was conducted. Among patients with medication errors, an average of 1.88 errors occurred per patient (SD 1.30; range 1–9). The most common types of errors are continued use of drugs with only ICU indications (28.4%), untreated conditions (19.4%), and unindicated drug therapy (11.9%) [6]. Brown examined the focus, structure, and purpose of the doctor's progress statement for patients transferred from the intensive care unit (ICU) to the hospital ward to find opportunities to improve communication interruptions. The medical records of adult patients who agreed to the doctor's transfer from the medical ICU to the hospital ward were analyzed. Using the mixed-effect linear regression model that illustrates the clustering within the hospital, the number, length, legibility, and content of the annotations in the care setting were counted and compared [7].

The innovation in this article is to establish a deeply trusted network model and compare the therapeutic effect of the intelligent processor 3D quality model in the ICU from the patient's clinic with other treatment methods in every respect. In view of the role of intelligent processor 3D quality model in patient treatment, the comparative results of experimental data were analyzed in more detail at different levels by collecting relevant experimental data. Finally, it was concluded that, under the test based on patients and experts, the experimental conclusions showed that the intelligent processor three-dimensional quality model was transferred in the ICU. It has high practical value in the treatment of patients.

## 2. Analysis Methods of Safety Influencing Factors and Management Countermeasures during the Transition Period

### 2.1. Transitional Mode

**Figure 1:**
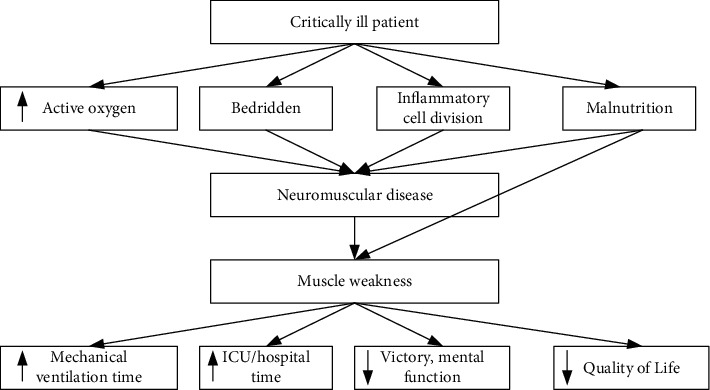
The hazards of immobilization in critically ill patients.

A critical/intensive patient is one whose clinical condition is unstable or potentially unstable and one or more organs or system functions are affected and may be life-threatening or potentially life-threatening [8]. Such patients often need to be concentrated in the intensive care unit (ICU) to receive systematic monitoring and treatment due to their serious illness and rapid changes. Recent survey data show that the number of critically ill patients in the US ICU reaches 4 million per year, and the ICU survival rate is as high as 80%–90% [9]. The number of critically ill patients admitted to China's ICU is increasing year by year, and the success rate of emergency and critically ill patients in the “third-class” hospitals is also ≥90% [10]. With the advancement of severe technology, the survival rate of ICU patients is increasing [11]. However, many surviving patients have suffered severe cognitive, physical, and psychological impairments due to critical illness and bed immobilization. The hazards of immobilization in critically ill patients are shown in Figure 1.

In today's society, the aging of the population is becoming more and more prominent, and due to the continuous improvement of living conditions and people's weak awareness of physical exercise, people generally have insufficient activity, which makes the elderly obese or overweight. The proportion has increased, and the accompanying basic diseases such as hypertension, diabetes, and hyperlipidemia have also increased year by year [12]. Due to long-term illness and lack of attention to the disease, some patients have inadequate control of the disease, causing complications such as the heart, kidneys, peripheral blood vessels, and nerves. This worsens the patient's condition. Multiple organ functions are involved in clinical practice. It is more and more common in treatment. Once the onset of disease occurs, the disease often deteriorates rapidly due to the chain reaction of the disease, and it is often necessary to enter ICU for treatment [13].

The discharge plan sets out transitional care. The transitional care model follows the discharge planning process, starting from the moment the patient is admitted to the hospital, including evaluation, arrangement, implementation, evaluation, discharge, follow-up after discharge, and other time sequence processes [14]. The key elements of transitional care are as follows: the overall coordinating role of transitional nurses in transitional care; assessment during admission and discharge planning; regular home visits and telephone contact for patients after discharge; establishing effective contact between the hospital and the community; paying attention to patients; providing active health education and support for patients, nurses, and families; assessing risk factors and improving patient conditions to reduce unnecessary hospitalization; effectively coordinating the communication between physicians, nurses, and multidisciplinary teams, including information transmission, patient preparation, and their treatment caregivers; and supporting self-management and expressing their needs [15, 16].

The purpose of paying attention to transitional care is to ensure patient safety and improve the quality of care [17]. Health education and effective communication and coordination of patients and caregivers are the focus of nurses' work during the transitional period of high-risk children. ① Coordinate the discharge preparation coordination meeting. The discharge team and family caregivers will discuss matters of attention and caregivers' concerns. For predischarge education, the meeting is usually presided over by a transitional nurse. ② Develop a nursing plan, assist family members to understand the disease problem, and review the implementation of the discharge guidance plan to ensure that patients and caregivers can understand the information and effectively implement the nursing plan. ③ Provide case management for patients and families. Before being discharged from the hospital, one of the important goals of transitional nurses is to encourage the patient's family to carry out nursing at home. ④ Provide professional clinical nursing services, such as stoma, duct care, oxygen therapy, and drug care. ⑤ Handover with community nursing staff.

The implementation of family-centered care in the ICU is an important part of the transitional care when the condition is getting better. Medical staff formulate personalized nursing measures for the patient and their family according to the situation of the patient and their family and actively communicate with their family members. Family members can actively participate in medical decision-making and directly participate in the patient's bedside care [18, 19].

### 2.2. Security Influence Factors

The traditional notion is that “making mistakes is shameful, and those who make mistakes should be blamed, insulted, criticized, and face even more severe punishment.” “Patient safety is primarily the responsibility of medical staff.” There is no hiding the fact that this concept impedes the application of modern security concepts. The modern concept of security believes that the existence of a culture of shame prevents people from admitting their mistakes and therefore makes people miss the opportunity to learn from their mistakes. The health system must abandon this culture. All managers and employees are responsible for the safety of patients, and nursing staff also play an important role in it, but they cannot replace the roles of other departments and personnel. Without the participation of other personnel in medical institutions, the safety goal cannot be fully achieved [20]. Establish a nonpunishable, convenient, and confidential error and related problem reporting system, monitor and analyze reported errors or problems, and provide feedback to the clinic. Hospitals can take strategic defensive measures to prevent errors and their consequences appear or mitigate. Errors that occur are one of the main measures to improve patient safety.

When calculating the safety influence factors, we use logistic regression algorithm to calculate them [21]. The logistic regression algorithm is actually the simplest algorithm in the neural network, which involves the loss function, which can also be called the log-likelihood function, and the larger the value of this function, the better.

(1) $$\begin{array}{c} \text{net}=\sum_{i=0}^{n}w_{i}x_{i}, \end{array}$$

(2) $$\begin{array}{c} o=\sigma \left(\text{net}\right) \end{array}$$

(3) $$\begin{array}{c} f\left(x\right)=\text{sigmoid}\left(w^{T}x+b\right). \end{array}$$

Now combine the linear function with the sigmoid function. Replace* z *to get *θ*^*T*^*x* the model algorithm function:

(4) $$\begin{array}{c} h_{\theta }\left(x\right)=g\left(\theta^{T}x\right)\\ =\frac{1}{1+e^{-\theta^{T}x}}. \end{array}$$

In the entire compound function, sigmoid is a fixed function with no parameters. So *θ* is the only parameter.

Our purpose of constructing the loss function is because we need to have a standard under which to find the best model that can best fit the training samples.

(1) The strategy function is
  (5) $$\begin{array}{c} {\left(h_{\theta }\left(x_{i}\right)\right)}^{y_{i}}{\left(1-h_{\theta }\left(x_{i}\right)\right)}^{1-y_{i}}. \end{array}$$
The larger the value, the better the model fitting the sample.
(2) Consider the impact of the sample on the model:
  (6) $$\begin{array}{c} \prod_{i=1}^{m}{\left(h_{\theta }\left(x_{i}\right)\right)}^{y_{i}}{\left(1-h_{\theta }\left(x_{i}\right)\right)}^{1-y_{i}}. \end{array}$$
(3) Construct the loss function of the logistic regression classifier:
  (7) $$\begin{array}{c} L\left(\theta \right)=\prod_{i=1}^{m}{\left(h_{\theta }\left(x_{i}\right)\right)}^{y_{i}}{\left(1-h_{\theta }\left(x_{i}\right)\right)}^{1-y_{i}}. \end{array}$$

If a certain value of the harness happens to make the loss function reach the maximum, then the model corresponding to this value is the best model.

The Korean style of the loss of the logistic regression classifier is to find the maximum value, and *L*(*θ*) is different to find the minimum value by the general loss function. The logarithm is as follows:

(8) $$\begin{array}{c} l\left(\theta \right)=\log\;L\left(\theta \right)\\ =\sum_{i=1}^{m}\left(y_{i}\log\;h_{\theta }\left(x_{i}\right)+\left(1-y_{i}\right)\log\left(1-h_{\theta }\left(x_{i}\right)\right)\right). \end{array}$$

After taking the logarithm, we call it the log-likelihood function. The derivation of the above formula is as follows:

(9) $$\begin{array}{c} \frac{\partial L\left(\theta \right)}{\partial \theta }=\sum_{i=1}^{n}y_{i}x_{i}-\sum_{i}^{n}\frac{e^{\theta^{T}x_{i}}}{1+e^{\theta^{T}x_{i}}}x_{i}\\ =\sum_{i=1}^{n}\left(y_{i}-\sigma \left(\theta^{T}x_{i}\right)\right)x_{i}. \end{array}$$

The iteration weight is as follows:

(10) $$\begin{array}{c} \theta_{j}=\theta_{j}+\alpha \sum_{i=1}^{m}\left(y_{i}-h_{\theta }\left(x^{\left(i\right)}\right)\right)x^{\left(i\right)}. \end{array}$$

The iterative process is not always convergent. Accuracy can measure the ratio of the number of correctly classified positive samples to all positive samples in a sample, and recall can measure the ratio of the number of correctly classified positive samples to all positive samples. Suppose the correctly classified set is *A*, and the wrong set is *B*:

(11) $$\begin{array}{c} \text{Precision}\left(A,B\right)=\frac{\left|A\cap B\right|}{\left|A\right|}, \end{array}$$

(12) $$\begin{array}{c} \text{Recall}\left(A,B\right)=\frac{\left|A\cap B\right|}{\left|B\right|}. \end{array}$$

Coverage refers to the proportion of the number of samples we selected in the population.

(13) $$\begin{array}{c} \text{Accuracy}=\frac{1}{n}\sum_{i=1}^{n}1\left({\hat{y}}_{t}=y_{t}\right). \end{array}$$

Support can measure the frequency of *AB* simultaneous occurrence. The frequency is small, indicating that *AB* has a small correlation, and vice versa; it indicates a strong correlation. Confidence measures whether or not *B* will appear when *A* appears, or its probability of appearing. A low degree of confidence indicates that the presence of *A* has little to do with whether *B* appears; on the contrary, the presence of *A* has a greater relationship with whether *B* appears.

(14) $$\begin{array}{c} \text{Support}\left(A⟶B\right)=P\left(A\cup B\right), \end{array}$$

(15) $$\begin{array}{c} \text{Confidence}\left(A⟶B\right)=P\left(B|A\right). \end{array}$$

*F*1-score is the harmonic average of precision rate and recall rate, which is closer to the smaller value of precision rate and recall rate.

(16) $$\begin{array}{c} F1=\frac{2\times \text{precision}\times \text{recall}}{\text{precision}+\text{recall}}. \end{array}$$

### 2.3. 3D Quality Model of Intelligent Processor

Build a 3D quality model with intelligent processing. Intelligent technology is an interactive dynamic virtual environment built by sensor technology, computers, and artificial intelligence that can affect human perception and make people feel like they are there [22]. Intelligent practice is the product of the rapid development of computer, Internet, information technology, and virtual reality technology [23]. This type of practice is separated from scientific practice and forms a new practice mode. The usual intelligent practice refers to the purposeful and two-way object-oriented perceptual activity that the subject performs on the object in an intelligent environment through digital media. Different from traditional practice, intelligent practice has the characteristics of real-time interaction, intelligent reality, and communication immersion. Its practice process is not restricted by external objective conditions, transcends the constraints of time and space, and can act on objective reality [24].

The application of smart technology and big data will be a general direction for future developments, but existing medical instruction rules and existing information systems will take longer to fully implement [25]. Virtual reality still has many challenges at the technical and business level, and issues such as capacity and performance issues and security limitations have yet to be resolved [26].

In the future, the application prospects of the combination of smart medical and blockchain technology are very broad, including important discoveries in the future about smart medicine, artificial intelligence, medical robots, blockchain technology, 3D printing technology, medical data, and biotechnology. These six technological innovations will completely change our traditional medical model. From the perspective of complexity and technical level, the automation of robotic processes is very accurate, and from the perspective of medical accuracy, it is a perfect combination of intelligence [27]. This development process requires the joint efforts of all personnel.

**Figure 2:**
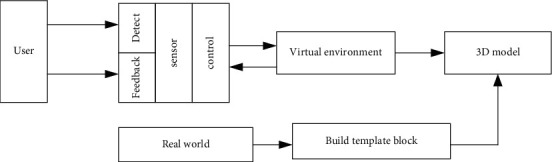
Intelligent processor 3D quality model technology composition.

Intelligent technology is different from traditional media. In an intelligent information environment, we are not only the publisher of information, but also the mediator of information dissemination and the recipient of information. This kind of media technology itself is an information field. There is no information center or authority in this field. Every entrance of intelligent reality is the receiver, relay, or sender of information. When we are in smart technology, we may be confronted with a “dehumanized” object. Perhaps this object is an object shaped by the subject's consciousness, but we can communicate with it like a stranger, from a certain level. In the above, we are our own communication, but the object is a materialized ideal object in our own imagination [28]. But unlike the split personality, under the technology of artificial intelligence, the object we conceive can become a dual existence that has both self-awareness and characteristics endowed by the subject. In this communication process, media technology not only creates the subject, but also acts as an intermediary and also serves as an object receiver. This new trinity communication method did not exist before the maturity of virtual reality technology [29]. This article combines intelligent technology into medical treatment, which can effectively detect the transfer of patients from ICU. The composition of intelligent technology is shown in Figure 2.

Due to many shortcomings and limitations of traditional sports rehabilitation therapy in clinical applications, people propose a more complete rehabilitation training system that can improve the subjective initiative of patients participating in treatment and improve the effectiveness of rehabilitation medicine. Therefore, the production of intelligent processor three-dimensional quality models is the future trends in medical treatment.

## 3. Safety Influencing Factors and Management Experiments of Patients Transferred from ICU during the Transition Period

### 3.1. Subjects

Cases were selected from the first diagnosed patients who visited our ICU from November 2019 to March 2020 and those who could be observed for more than 1 month after treatment. The following requirements must be met at the same time.

#### 3.1.1. Selection Criteria

Selection criteria include the following:

1. Patients transferred from ICU
2. Those 35–60 years old
3. Those able to cooperate with various inspections

We combined the survey results to carry out targeted prevention and treatment-related knowledge and clinical practice training in various forms, different contents, and time periods and explore the best training methods suitable for the current situation in our province, so as to improve the relevant clinical nursing staff in our province, and the theoretical level of knowledge and the ability of clinical practice to improve the level of nursing in our province, to improve the effect of patients' recovery and management during the transition period, and to provide help to reduce the incidence of accidents.

#### 3.1.2. Exclusion Criteria

Exclusion criteria include the following:

1. Hypertensive emergency/hypertensive subemergency/secondary hypertension/refractory hypertension/white coat hypertension
2. Diabetes, hyperlipidemia, history of malignant tumors, liver cirrhosis and/or liver failure, history of narcotic drug abuse, and significant lifestyle changes in the past 12 months
3. All factors that can increase the mortality rate, such as coronary heart disease, various arrhythmias, various cardiomyopathy and valvular diseases, severe heart failure, cardiogenic shock, pericardial tamponade, pacemaker implantation, pericardial inflammation, chronic obstructive pulmonary disease, pulmonary embolism, and people with obvious infection
4. Combined with severe liver and kidney damage and primary diseases related to the hematopoietic system and endocrine system
5. Patients with acute inflammation: hs-CRP > 10 mg/L or white blood cell count > 10 × 10^9^/L
6. Women during pregnancy or lactation
7. Those who are allergic to drugs
8. Those with severe sleep disorders, mental disorders, and unwillingness to cooperate
9. Being a participant in other clinical trials at the same time.

After that, the statistical data is classified and analyzed, and processing is simulated through computer software.

### 3.2. Baseline Data

Baseline data include the following:

1. Data content: collect general data of the patient, such as age, gender, and cause of injury, and collect the total operation time, ICU resuscitation time, intraoperative blood loss, hospital stay, death, and complications of the patient during treatment
2. Collection method: query the electronic medical record system of the city's first hospital, find qualified patient information based on the patient's clinical diagnosis and the above screening criteria, borrow patient medical records in the medical record room, view patient-related information, and collect patient-related information

### 3.3. Patient Data Monitoring

All test takers choose the same portable cuff portable sphygmomanometer that has passed the device certification test procedure. This blood pressure monitoring is carried out by standardized training personnel, and the precautions shall be explained to the examinee during the measurement process (try to keep the upper arm still and avoid compression of the cuff and high-intensity exercise). The subject needs to continuously monitor blood pressure for at least 24 hours, and the frequency is set as follows: day (6:00–22:00) 30 min and night (22:00–6:00) 60 min. During this period, the sphygmomanometer will automatically inflate and record the corresponding blood pressure value once. The examiner calculates the average value of systolic and diastolic blood pressure for each period (including 24 h, day and night) based on the blood pressure value. If the effective blood pressure value detected within 24 hours is less than 80% of the blood pressure value that should be obtained or the data is missing for more than 2 hours continuously, the blood pressure test needs to be repeated.

### 3.4. Statistics

The data in this article are statistically analyzed using SPSS20.0 statistical software, and the measurement data are represented by xs±. If the data conform to the normal distribution and homogeneity of variance, the general *t*-test and analysis of variance are used. If the data do not conform to the normality, try variable transformations or use nonparametric tests.

## 4. Safety Influencing Factors and Management Experiments for Patients Transferred from ICU during the Transition Period

### 4.1. Comparison of Basic Clinical Data Characteristics of the Study Population

The *t*-test was performed on the basic clinical data indicators of the patients transferred from the ICU during the transition period. The statistical results are shown in Table 1: gender ratio, age, body mass index (BMI), blood sugar, and total cholesterol (TC). There was no significant difference in triglyceride (TG), high-density lipoprotein cholesterol (HDL-C), low-density lipoprotein cholesterol (LDL-C), creatinine, uric acid, and blood pressure levels (*P* > 0.05), as shown in Table 1.

As we can see, patients are not significantly associated with age, gender, blood glucose, calcium, phosphorus, triglycerides, cholesterol, or low-density lipoprotein (*P* > 0.05), but with body mass index, systolic blood pressure, diastolic blood pressure, and white blood cell count. There is a relationship and sensitive C-reactive protein. It is negatively correlated with carotid IMT (*P* < 0.05) and positively correlated with high-density lipoprotein (*P* < 0.01), as shown in Table 2.

After adjusting for factors such as age, body mass index, systolic blood pressure, diastolic blood pressure, white blood cell count, high-sensitivity C-reactive protein, blood sugar, calcium, phosphorus, triglycerides, cholesterol, and high-density lipoprotein and low-density lipoprotein, the results were still consistent with the observation group. Independent negative correlation, partial correlation coefficient is −0.847 (*P* < 0.01). We compare the time-domain indicators of heart rate variability and the average heart rate machine of each group of patients, as shown in Table 3.

As shown in Table 3, compared with the control group, the observation group's SDNN decreased from 131.51 ± 8.48 ms to 98.63 ± 4.24 ms, SDANN decreased from 130.63 ± 8.29 ms to 95.25 ± 5.02 ms, rMSSD decreased from 31.89 ± 3.72 ms to 14.22 ± 1.79 ms, pNN50 decreased from 11.96 ± 0.88% to 8.02 ± 0.38%, and 24hmHR increased from 69.10 ± 1.20 times/min to 78.56 ± 1.45 times/min (*P* < 0.05). The blood pressure variability between patients is shown in Table 4.

### 4.2. Changes in Patient Care

**Figure 3:**
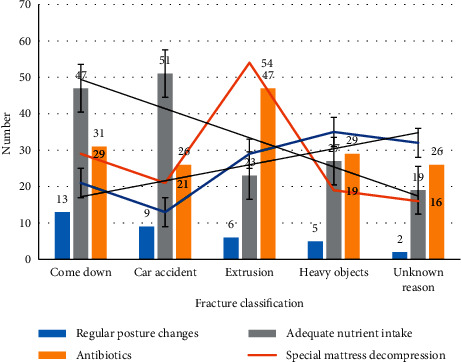
Number of people treated with pressure ulcers by different methods.

For patients transferred from the ICU, the current treatment methods include regular posture changes, special matters decompression, adequate nutrient intake, antibiotics, and the three-dimensional quality model of the intelligent processor used in this article. We make statistics on the current treatment population. The details are shown in Figure 3.

From Figure 3, we can see that, at this stage, because the patient does not understand the situation, the intelligent processor 3D quality model is not the first choice, because this method requires more steps and costs. It can be seen from the figure that the main reason for patients transferred out of ICU is mainly due to trauma. The hemodynamics of the patients are extremely unstable when they are admitted to the hospital. In addition, the severe trauma caused the fracture to cause damage to the surrounding organs, skin and soft tissues, and main blood vessels.

**Figure 4:**
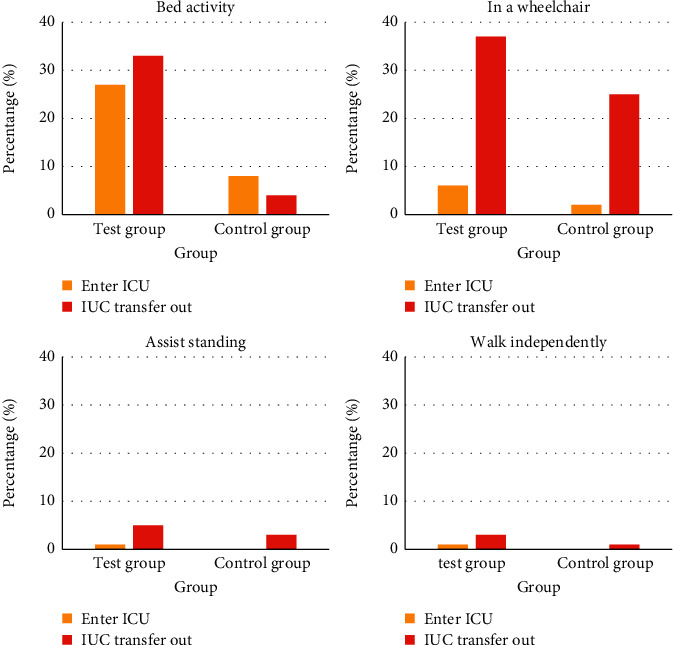
Changes before and after patient transfer from ICU.

After treatment, the active exercise of patients in the experimental group transferred from the ICU from the bed was significantly higher than that in the control group (*P* < 0.05). Compared with the control group, patients in the experimental group had a rising trend when they were transferred from ICU, assisted in standing/walking, and walking independently, but the difference was not statistically significant (*P* > 0.05). The details are shown in Figure 4.

**Figure 5:**
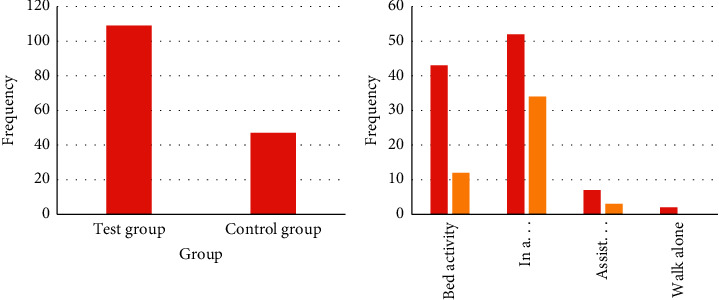
Comparison of patient exercise frequency.

We have counted the patient's exercise frequency and understood the number of exercises of patients transferred from the ICU, so as to judge the physical recovery of the patient after transferring. The specific situation is shown in Figure 5.

As shown in Figure 5, the number of active exercises in the test group (109 times) was significantly higher than that in the control group (49 times) (*P* < 0.05). Compared with the control group, the number of active exercises, wheelchairs, and standing/walking assistance in the experimental group increased significantly, and the difference was statistically significant (*P* < 0.05). In addition, in ICU walking independently, the test group increased compared with the control group, but the difference was not statistically significant (*P* > 0.05).

### 4.3. Comparison of Influencing Factors

**Figure 6:**
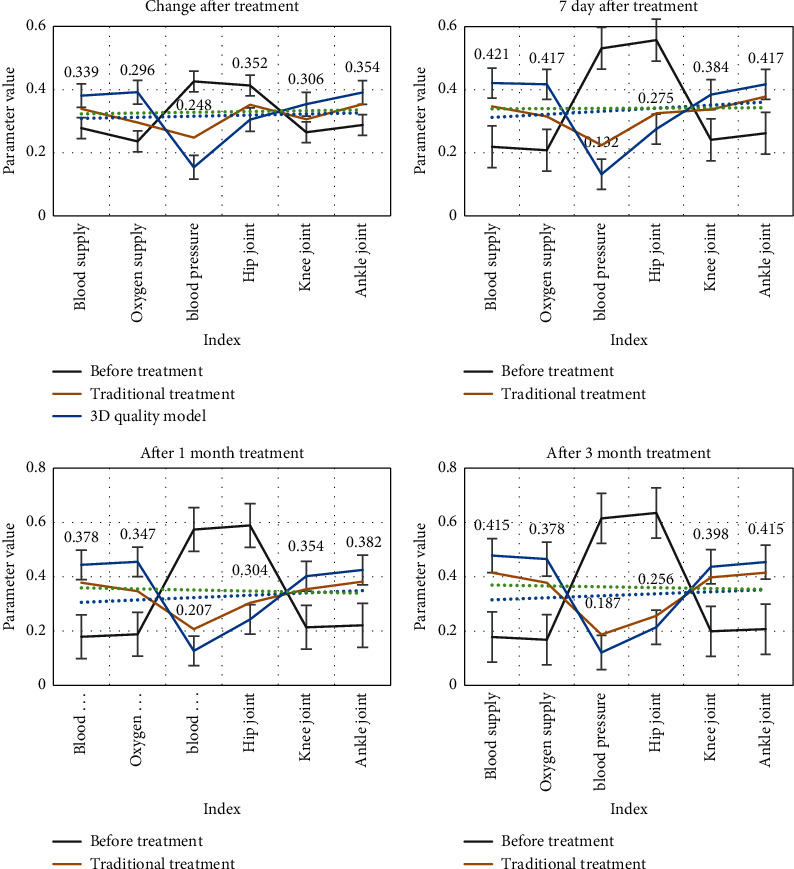
Before and after patient treatment.

We look at the relevant parameters before and after the treatment of the patient and compare the parameters before and after the treatment to understand the treatment effect, as shown in Figure 6.

From Figure 6, we can see that, after treatment, the patient's physical skills have improved significantly. Among them, the patient's blood supply and oxygen supply have been significantly improved, and their own mobility has been significantly improved. After 3 months of treatment, the traditional treatment method has a clear comparison with the treatment effect based on the intelligent processor 3D quality model in this article. The treatment effect based on the intelligent processor 3D quality model is significantly better than the traditional treatment effect, and the patient's physical condition is obviously improved.

**Figure 7:**
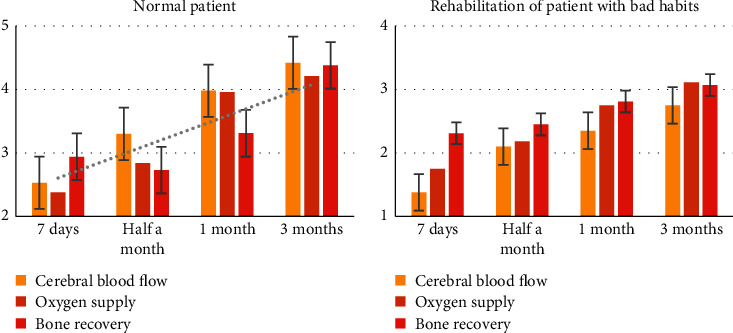
Comparison of patients' prognosis and rehabilitation.

We compare the transitional prognosis of patients with normal schedules and patients with bad habits, as shown in Figure 7.

From Figure 7, we can see that, compared with patients with normal work and rest, patients with poor work and rest have much lower recovery speed and efficiency. This shows that good living habits are extremely important for the prognosis and recovery of patients, and attention should be paid during the transition period of patients. The patient's living habits should maintain a good schedule.

## 5. Conclusion

With the development of economy, society, and medicine, “improving patient prognosis and outcome” has become an increasingly important issue in the field of intensive care in various countries. For patients transferred from the ICU, the intelligent processor-based three-dimensional quality model can reduce inflammation, prevent insulin resistance and microvascular dysfunction, improve the quality of life and functional status of critically ill patients, and provide ventilation, thereby reducing time and reducing ICU hospital stay. Reducing mortality and treatment costs also requires reasonable resource support, daily dynamic assessment, and real-time adjustment and optimization of care plans to achieve a patient-centered personalized rehabilitation prognosis. Of course, the research in this article also has its shortcomings. Due to hardware equipment and limited learning time, only some patients in the city are collected. This sample has certain limitations and will inevitably deviate from the actual situation. In the future, we hope that more researchers can focus on the application of intelligent processor 3D quality models in the treatment of patients transferred from ICU, so that they can more accurately detect patients transferred from ICU in the future.

## Figures and Tables

**Table 1:** Comparison of data between groups of patients.

	Control group	Observation group	Plaque formation group
Male/female (number of cases)	13/9	17/19	18/12
Age	47.25 ± 11.72	52.15 ± 11.35	51.93 ± 11.23
BMI (kg/m^2^)	25.17 ± 1.63	25.06 ± 1.53	26.12 ± 1.57
Blood sugar (mmol/L)	5.51 ± 1.03	5.47 ± 1.12	5.33 ± 1.24
Uric acid (umol/L)	323.23 ± 81.42	329.02 ± 82.55	340.91 ± 92.16
Creatinine (umol/L)	78.35 ± 21.33	79.13 ± 23.27	79.98 ± 31.16
TC (mmol/L)	5.63 ± 0.88	5.72 ± 0.91	5.68 ± 1.21
TG (mmol/L)	3.67 ± 0.46	2.91 ± 0.84	2.81 ± 0.52
HDL (mmol/L)	2.33 ± 0.17	2.28 ± 0.12	2.24 ± 0.14
LDL (mmol/L)	4.04 ± 0.70	3.91 ± 0.21	4.24 ± 0.69

**Table 2:** Correlation results of each indicator.

	BMI	SBP	DBP	White blood cell count	hsCRP	HDL	IMT
Correlation coefficient	−0.238	−0.425	−0.211	−0.272	−0.208	0.325	−0.832
*P*	0.014	0.005	0.002	0.006	0.032	0.002	0.013

**Table 3:** Comparison of time-domain indicators of heart rate variability and average heart rate between groups of patients.

Project	Control group	Observation group	Plaque formation group
SDNN (ms)	131.51 ± 8.48	98.63 ± 4.24	55.25 ± 6.64
SDANN (ms)	130.63 ± 8.29	95.25 ± 5.02	49.01 ± 3.99
rMSSD (ms)	31.89 ± 3.72	14.22 ± 1.79	4.11 ± 0.59
pNN50 (%)	11.96 ± 0.88	8.02 ± 0.38	5.46 ± 0.27
24hmHR (times/min)	69.10 ± 1.20	78.56 ± 1.45	89.48 ± 1.82

**Table 4:** Blood pressure variability between patients.

Project	Control group	Observation group	Plaque formation group
24hSSD (mmHg)	10.22 ± 3.01	13.88 ± 3.56^*∗*^	16.40 ± 3.23
24hDSD (mmHg)	8.32 ± 3.99	10.19 ± 3.45^*∗*^	13.78 ± 3.09
dSSD (mmHg)	10.24 ± 2.36	13.35 ± 2.55^*∗*^	15.98 ± 2.80
dDSD (mmHg)	9.41 ± 3.48	11.82 ± 3.76^*∗*^	13.81 ± 3.99
nSSD (mmHg)	9.96 ± 4.12	12.13 ± 4.09^*∗*^	14.18 ± 4.18
nDSD (mmHg)	8.01 ± 3.31	10.36 ± 3.26^*∗*^	11.66 ± 3.48

## Data Availability

No data were used to support this study.
